# Potassium is a trigger for conformational change in the fusion spike of an enveloped RNA virus

**DOI:** 10.1074/jbc.RA118.002494

**Published:** 2018-04-20

**Authors:** Emma K. Punch, Samantha Hover, Henry T. W. Blest, Jack Fuller, Roger Hewson, Juan Fontana, Jamel Mankouri, John N. Barr

**Affiliations:** From the ‡ School of Molecular and Cellular Biology and; ¶ Astbury Centre for Structural Molecular Biology, University of Leeds, Leeds, LS2 9JT, United Kingdom and; the § National Infection Service, Public Health England, Porton Down, Salisbury SP4 0JG, United Kingdom

**Keywords:** virus entry, fusion protein, electron tomography, conformational change, potassium transport, ion channel, virus structure

## Abstract

Many enveloped viruses enter cells through the endocytic network, from which they must subsequently escape through fusion of viral and endosomal membranes. This membrane fusion is mediated by virus-encoded spikes that respond to the dynamic endosomal environment, which triggers conformational changes in the spikes that initiate the fusion process. Several fusion triggers have been identified and include pH, membrane composition, and endosome-resident proteins, and these cues dictate when and where viral fusion occurs. We recently reported that infection with an enveloped bunyavirus requires elevated potassium ion concentrations [K^+^], controlled by cellular K^+^ channels, that are encountered during viral transit through maturing endosomes. Here we reveal the molecular basis for the K^+^ requirement of bunyaviruses through the first direct visualization of a member of the Nairoviridae family, namely Hazara virus (HAZV), using cryo-EM. Using cryo-electron tomography, we observed HAZV spike glycoproteins within infectious HAZV particles exposed to both high and low [K^+^], which showed that exposure to K^+^ alone results in dramatic changes to the ultrastructural architecture of the virion surface. In low [K^+^], the spikes adopted a compact conformation arranged in locally ordered arrays, whereas, following exposure to high [K^+^], the spikes became extended, and spike–membrane interactions were observed. Viruses exposed to high [K^+^] also displayed enhanced infectivity, thus identifying K^+^ as a newly defined trigger that helps promote viral infection. Finally, we confirmed that K^+^ channel blockers are inhibitory to HAZV infection, highlighting the potential of K^+^ channels as anti-bunyavirus targets.

## Introduction

The order Bunyavirales represents the largest group of enveloped segmented negative-stranded (SNS)^2^ RNA viruses, and many are associated with serious disease of humans, animals, and plants. All bunyaviruses possess small (S), medium (M), and large (L) RNA segments that exhibit a similar coding strategy; their S segments encode an RNA-binding nucleoprotein (N), their M segments encode a polyprotein precursor that is cleaved to form G_n_ and G_c_ envelope-associated glycoproteins, and their L segments encode the viral RNA-dependent RNA polymerase. Additional nonstructural proteins, NSs and NSm, are often expressed from S and M segments, either by accessing alternate reading frames or by expression of additional mRNAs using an ambisense transcription strategy (1). The order includes over 500 named isolates classified into nine families, with four of these including members that cause serious human disease; namely, the Nairoviridae, Peribunyaviridae, Hantaviridae, and Phenuiviridae (2).

All members of the Nairoviridae family are arboviruses, including Crimean–Congo hemorrhagic fever virus (CCHFV), which is an emerging threat because of the expanding habitat of its tick host (3). CCHFV is the causative agent of a lethal hemorrhagic fever in humans, with case fatality rates of over 60% in certain outbreaks (4). There is no vaccine or therapy to prevent or treat CCHFV-mediated disease, and consequently, CCHFV is one of a small group of human pathogens classified in hazard group 4. Hazara virus (HAZV) is closely related to CCHFV, and they share the same CCHFV serogroup and high structural homology (5, 6), although HAZV has not been documented to cause human disease and is currently classified within hazard group 2.

The nairovirus replication cycle begins with cell attachment followed by entry predominantly through clathrin-mediated endocytosis (7–9), whereas other bunyaviruses have been reported to use clathrin-independent entry mechanisms (10). A common hurdle for all bunyaviruses is escape of the three RNA segments from virions captive within endosomes, which requires fusion of the viral and endosomal membranes, a process orchestrated by the G_n_ and G_c_ glycoproteins located on the virion exterior as heteromultimeric spikes. All available evidence suggests that bunyavirus fusion is mediated by the G_c_ protein, and the solution of G_c_ crystal structures from members of the Phenuivirus (11–13) and Hantavirus groups (14–16) shows that they possesses a common class II fusion protein fold also shared by spike glycoproteins of flaviviruses (17), alphaviruses (18), and togaviruses (19).

The fusion process has been characterized for many SNS RNA viruses, and a general model suggests that viral fusion proteins must first be primed before they can be triggered to begin the fusion process. The canonical priming step involves proteolytic cleavage, whereas fusion triggers include low pH, interaction with cellular receptors, or an encounter with specific endosomal lipids (20, 21). Many fusion proteins take advantage of the changing biochemical environment of the endosomal system so that they respond with conformational changes and become fusion-active only in specific compartments where precise biochemical cues are encountered.

For nairoviruses, biogenesis of the fusion machinery is complex and involves multiple cellular proteases, including the SKI-1/S1P serine protease, to eventually generate G_c_ and G_n_ moieties (22). However, the events that trigger the fusion protein to adopt its fusogenic conformation are poorly understood.

Recently, using the model bunyavirus Bunyamwera virus (BUNV), we showed that pharmacological inhibition of endosomal K^+^ channels blocked BUNV entry by reducing K^+^ accumulation in the endosomal system. In the absence of necessary [K^+^] in endosomes, infecting virions were trafficked to lysosomes, where they became inactivated by low pH (23). In contrast, *in vitro* exposure of BUNV to elevated [K^+^] at pH 6.3 to mimic the endosome environment resulted in increased infectivity (23). This is relevant in the context of viral entry and fusion, as all cells exhibit a K^+^ gradient that ranges from 5 mm in extracellular spaces to 140 mm in the cytosol. Furthermore [K^+^] increases with passage through the endocytic pathway, concomitant with a drop in pH (24). Although this work revealed [K^+^] as a newly identified biochemical cue required for efficient infectivity, the identity of the viral component that responds to the K^+^ was unknown, as was the nature of the underlying mechanism of enhanced infection.

Here we demonstrate that entry of HAZV, like BUNV, is also influenced by [K^+^], showing that K^+^ enhances infectivity across multiple families within the Bunyavirales order. We further report the first visualization of any nairovirus using cryo-EM, which revealed the HAZV ultrastructural characteristics and surface glycoprotein architecture in high detail. By performing cryo-electron tomography (cryo-ET) of HAZV and subtomogram averaging (STA) of HAZV spikes, we show that exposure of virions to elevated [K^+^] alone is associated with dramatic spike conformational changes and, furthermore, promotes interactions with membranes. We propose that these conformational changes and membrane interactions occur during the HAZV entry pathway and initiate virus fusion within endosomal compartments in which the [K^+^] trigger is present. Finally, we show that the cellular K^+^ channels that mediate endosomal K^+^ influx are a druggable target that can be targeted to block HAZV infectivity. Taken together with our previous findings (25) showing that multiple bunyaviruses require elevated [K^+^] for entry, we suggest that the [K^+^] trigger is a common feature of bunyaviral entry and that cellular K^+^ channels represent a new target for the development of antiviral molecules that broadly impede bunyavirus growth.

## Results

### Exposure of HAZV to elevated [K^+^] increases infectivity

Our previous results showed that infectivity of the prototypic bunyavirus BUNV was enhanced by *in vitro* exposure of purified virions to elevated [K^+^] within the range of 20–140 mm (23). These [K^+^] values represent the range found within the lumen of late endosomes, compared with physiological levels of K^+^ present within the extracellular milieu, of around 5 mm (23). Exposure to elevated [K^+^] accelerated the entry of BUNV into cells, reducing the time taken for onset of viral gene expression, as measured by production of BUNV N protein. We proposed that exposure of virions to elevated [K^+^] within specific endosomal compartments represented a biochemical cue that was a requirement for efficient virus infectivity.

**Figure 1.**
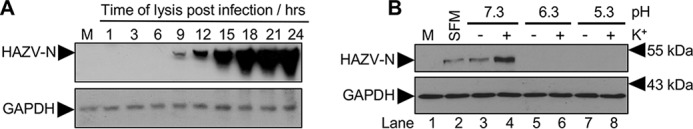
**HAZV infectivity can be increased by exposure to elevated [K^+^].** *A*, time course of HAZV multiplication in A549 cells infected at an m.o.i. of 0.1 or mock-infected (*M*) as assessed by HAZV N protein production, detected by Western blotting with anti-HAZV N antiserum alongside GAPDH loading controls. Lanes are labeled with time of harvest after infection in hours. A long exposure of the Western blotting is shown to reveal early N production. *B*, HAZV was exposed *in vitro* to either low (5 mm, −*K*^+^) or high (140 mm, +*K*^+^) relative [K^+^] at a pH of 5.3, 6.3, or 7.3. After incubation, [K^+^] were diluted by addition of SFM (K^+^ concentration, 5.3 mm), and K^+^-exposed virions were used to infect A549 cells at an m.o.i. of 0.1 for 18 h. Cells were also mock-infected and incubated with virus incubated in SFM alone. The abundance of HAZV N protein expression was assessed by Western blot analysis as described for *A*, with a representative blot shown here.

Here we examined whether HAZV infectivity could also be increased by exposure to [K^+^]. To do this, HAZV was exposed *in vitro* to [K^+^] that were either low (5 mm) or high (140 mm) at a pH of either 5.3, 6.3, or 7.3. After incubation, high K^+^ concentrations were diluted by addition of cell growth medium ([K^+^] = 5 mm), and K^+^ treated virions were used to infect A549 cells at an m.o.i. of 0.1 for 18 h. The abundance of HAZV N protein expression was assessed by Western blot analysis using anti-HAZV N antiserum, to serve as a measure of virus multiplication, with the 18-h time point chosen because of the abundant N expression (Fig. 1*A*).

HAZV particles exposed to either pH 5.3 or pH 6.3 failed to express detectable levels of N at the 18-h time point, irrespective of whether [K^+^] was high or low (Fig. 1*B*, *lanes 5–8*), suggesting that virions exposed to low pH conditions were functionally impaired for one or more early stages of the replication cycle. In contrast, HAZV particles exposed to pH 7.3 were infectious, with N protein detected for virions exposed to high or low [K^+^] (Fig. 1*B*, *lanes 3* and *4*) or incubated in serum-free medium (SFM; Fig. 1*B*, *lane 2*). However, exposure of virions to [K^+^] of 140 mm at pH 7.3 resulted in enhanced infectivity, with a 10-fold increase in the abundance of N protein expression at 18 h after infection, as determined by densitometry of three independent Western blotting analyses (Fig. 1*B*, *lane 4*). These results show that HAZV infectivity is increased in elevated [K^+^], similar to our previous findings with BUNV (23), which opens the possibility that other members of the Bunyavirales order may also respond to [K^+^] in the same way.

### Purification and visualization of HAZV by cryo-EM

To better understand the mechanism through which [K^+^] increases infectivity, we sought to visualize HAZV particles using cryo-ET. We reasoned that the molecular mechanism through which [K^+^] influenced infectivity likely involved the induction of conformational changes in one or more viral component(s). As bunyavirus particles do not possess ion-permeable channels that would permit K^+^ influx to the virion interior (25), we hypothesized that the most likely component to exhibit conformational changes were the surface-exposed G_n_ and G_c_ glycoproteins.

**Figure 2.**
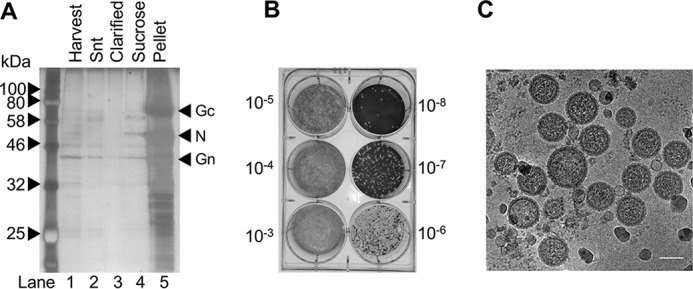
**Purification and cryo-EM of HAZV particles.** *A*, HAZV was harvested from infected cell supernatant (*Snt*), which was clarified and then pelleted by centrifugation through a sucrose cushion, and analyzed by SDS-PAGE. *B*, resuspended HAZV was titered on SW13 cells. *C*, HAZV was vitrified on carbon-backed grids for cryo-EM analysis, which revealed a pleomorphic virion morphology, with viral glycoprotein spikes visible around the perimeter. *Scale bar* = 100 nm.

Before applying cryo-ET to investigate this possibility, we first optimized HAZV purification and vitrification. Secreted HAZV particles were purified from BHK cell supernatants by ultracentrifugation through a 20% sucrose cushion. The resuspended pellet was assessed for overall protein concentration and purity by PAGE with silver staining (Fig. 2*A*, *lane 5*). Purified virus was tested for infectivity by plaque assay, which yielded a titer of ∼10^9^ pfu/ml.

Direct observation using cryo-EM revealed that HAZV particles possess a pleomorphic morphology with some variation in virion shape and dimensions (Fig. 2*C*). HAZV particles were mostly near-spherical, although virions with oval and elongated morphologies were also evident (data not shown). Previous studies have shown that the gross morphology of bunyaviruses ranges between icosahedral symmetry as a T = 12 lattice in the case of Rift Valley fever virus (RVFV) (26–28) to a highly pleomorphic morphology in the case of Tula hantavirus (29), with both tubular and spherical particles being observed. The morphology of peribunyaviruses BUNV and La Crosse virus are intermediate to these extremes (30, 31), being near spherical, and with some variation in particle diameter. Our observations suggest that HAZV does not possess icosahedral symmetry and instead is pleomorphic. HAZV virions possessed pointed projections around their perimeter and formed a mostly continuous electron-dense ring that was concentric with the lipid bilayer.

### Exposure to elevated [K^+^] induces conformational changes in HAZV glycoprotein spikes

With purification and vitrification parameters optimized, we next performed cryo-ET to directly observe the architecture of the HAZV envelope glycoproteins when treated with high or low [K^+^], again at a constant pH of 7.3. By choosing these defined conditions, we were able to directly correlate the cryo-ET observations with HAZV infectivity characteristics (Fig. 1*B*, *lanes 3* and *4*).

**Figure 3.**
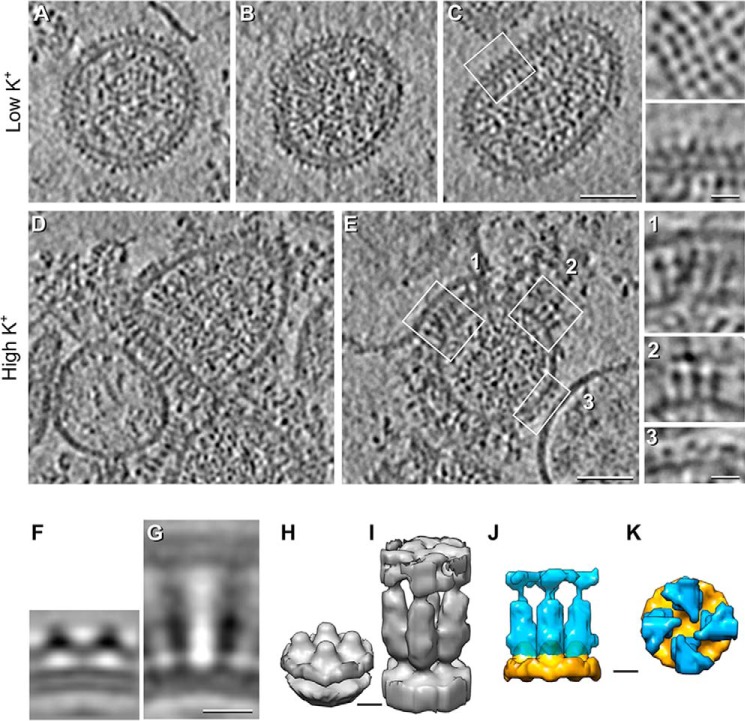
**Cryo-ET and STA of HAZV virions treated with low or high [K^+^] at pH 7.3.** *A–E*, central tomographic sections of HAZV virions. *A–C*, low [K^+^] (5 mm). A continuous glycoprotein array around the viral envelope is evident. The 4-fold arrangement of the spikes is shown in a tangential section of a HAZV virion (*C*, *top inset*). Magnification of the *squared region* in *C* is shown in the *bottom inset. D* and *E*, high [K^+^] (140 mm). Changes in [K^+^] resulted in extension of the glycoprotein spikes (*e.g. E*, *insets 1* and *2*) and interactions with adjacent membranes (*e.g. E*, *inset 1*) co-purified with HAZV virions. *E*, *inset 3*, shows control-like spikes in high [K^+^]–treated HAZV virions. *F–K*, STA of HAZV spikes: Sagittal sections (*F* and *G*) and isosurface (*H* and *I*) rendering of HAZV spikes at low (*F* and *H*) and high (*G* and *I*) [K^+^]. *J* and *K*, superimposed isosurface rendering of low (*orange*) and high (*blue*) [K^+^]–treated segmented spikes as seen from the side (*J*) and top (*K*). Although the high K^+^ average is ∼3 times longer than the low K^+^ average (*J*), the low K^+^ average shows a continuous density parallel to the viral envelope that is absent in the high K^+^ average (*K*). *Scale bars* = 50 nm in *A–E*, 10 nm in *F* and *G*, and 5 nm in *H–K*.

Single-axis tilt series were collected, which allowed the 3D tomographic reconstruction of multiple HAZV particles (Fig. 3). In agreement with the 2D images shown in Fig. 2*C*, HAZV virions exposed to low [K^+^] conditions were highly pleomorphic and possessed short surface projections ∼8 nm in height that represent the G_n_ and G_c_ heterodimer spikes (Fig. 3, *A–C*). Visualization of the virion surface revealed near-complete coverage with glycoprotein spikes, which formed locally ordered patches that appeared like a lattice, with similar spacing between adjacent electron-dense projections (Fig. 3*C*, *insets*). Electron density within the virion interior was evident and was likely attributable to the segmented HAZV RNA genome in association with the HAZV N protein. However, the structural characteristics of these ribonucleoprotein molecules were unresolved, likely because of their heterogeneous orientation within the virion interior.

In contrast, HAZV virions exposed to high [K^+^] exhibited dramatic changes to the peripheral glycoprotein spikes compared with the low [K^+^]–treated viruses (Fig. 3, *D* and *E*). First, most of the glycoprotein spikes exhibited an elongated appearance (Fig. 3*E*, *insets 1* and *2*). The elongated spikes were positioned perpendicular to the envelope and thus were maximally extended away from the virion surface. In many cases, the elongated spikes appeared wide at the tips and were seen to interact with membranes that had co-purified with the virions (Fig. 3*E*, *inset 1*). In most cases where these spikes were interacting, the target membranes were noticeably distorted and adopted a curved morphology that coincided with the curvature of the adjacent virion (Fig. 3*E*, *inset 1*). Second, many regions of the virion envelope appeared devoid of spikes, suggesting that some spikes had been shed, and in addition, the previously identified regions of locally ordered surface spikes observed under low K^+^ conditions were less evident (Fig. 3*E*, *inset 3*). Taken together, these observations show that elevated [K^+^] elicits architectural changes to the virion glycoprotein spikes and virion exterior and that these changes favor the interaction of the elongated spikes with cellular membranes.

**Figure 4.**
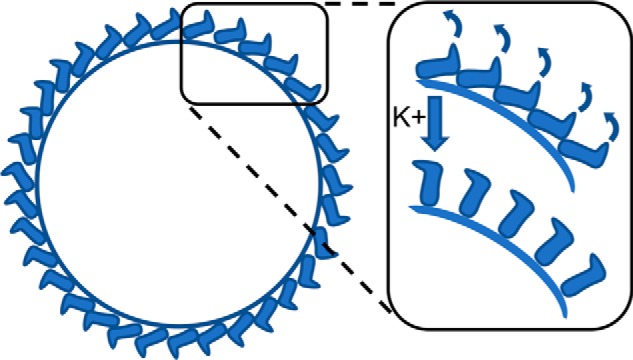
**Model describing the HAZV glycoprotein rearrangements after exposure to high K^+^.**

We performed STA of spikes from virions exposed to low [K^+^] at pH 7.3 using 4-fold symmetry (as 4-fold symmetry for HAZV spikes is suggested from top sections of HAZV particles; Fig. 3*C*, *top inset*), which allowed reconstruction of the spike ectodomain at a resolution of 25 Å. The side view revealed a compact arrangement of adjacent electron dense spikes lying close to the envelope surface (Fig. 3*F*). These spikes were arranged in a continuous layer of tetrameric complexes with a maximum height of 8 nm from the viral membrane. Individual protomers appeared as inverted “V” densities separated by ∼7.5 nm (Fig. 3, *F* and *H*). Under high [K^+^], the continuous layer is disrupted, and the spikes extend, becoming ∼22 nm tall (Fig. 3, *G* and *I*). Of note, the calculated volumes (low K^+^/high K^+^) have a ratio of 1.0:1.3 (1.1 × 10^6^ Å^3^ for the control average *versus* 1.4 × 10^6^ Å^3^ for the high K^+^ average). Therefore, the total volume of the spikes is similar under both conditions (Fig. 3, *J* and *K*), suggesting that the differences in the averages represent conformational changes of G_c_ and/or G_n_. We propose a model in which K^+^ mediates domain rearrangements of the G_n_/G_c_ ectodomain (Fig. 4) that permits spike extension and also interaction with a target membrane. In the context of virus entry, it is difficult to escape the conclusion that elevated [K^+^] within endosomal compartments represents a biochemical trigger that results in virion spike rearrangements that subsequently lead to fusion.

### HAZV multiplication is prevented by cellular K^+^ channel blockade

We recently showed for the first time that cellular K^+^ channels are required during entry of BUNV to permit K^+^ influx and maintenance of the endosomal [K^+^] gradient. Based on this premise and the increased infectivity of HAZV in high [K^+^] (Fig. 1*A*), we reasoned that blockade of endosomal K^+^ channels would be detrimental to HAZV infectivity, as endosomes would not reach the required [K^+^] to induce the necessary virion spike conformational changes. In this scenario, HAZV particles would fail to fuse with endosomes because of insufficient K^+^, and instead virions would be trafficked to later endosomal compartments and lysosomes, where the low pH would likely render the particles noninfectious.

**Figure 5.**
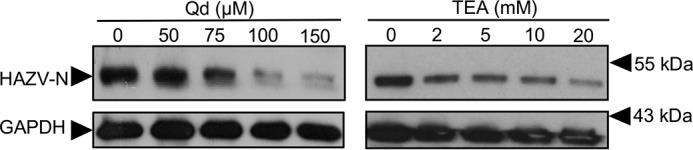
**HAZV multiplication can be blocked by broad-spectrum inhibition of cellular K^+^ channels.** Representative western blots of A549 lysates following pretreatment with the indicated channel blockers (TEA, and Qd) prior to infection with HAZV (m.o.i. of 0.1). After 24 h, cell lysates were probed by Western blotting with sheep anti-HAZV N serum and GAPDH as a loading control. No-treatment controls were included for each inhibitor.

Previously, we showed that the broad-spectrum K^+^ channel–blocking compound tetraethylammonium (TEA) dramatically reduced HAZV replication (27). Here we extended this work by demonstrating that HAZV replication can be inhibited by both TEA and also quinidine (Qd), which is another well-characterized broad spectrum K^+^ channel–blocking agent. A549 cells were pretreated with either TEA or Qd, followed by infection with HAZV at an m.o.i. of 0.1 (Fig. 5). Measurement of HAZV N protein expression at the 24-h time point showed reduced HAZV growth at nontoxic concentrations of both channel blockers, with Qd in particular resulting in over 90% reduction in N protein expression. The inhibition of HAZV infection under K^+^ channel blockade further supports the critical role of [K^+^] in the HAZV entry pathway. This inhibition coincides with that recently reported for both Schmallenberg virus (SBV) and BUNV (25), the prototypic Bunyavirales order member, suggesting that cellular K^+^ channels may represent an order-wide target for the development of anti-bunyaviral compounds.

## Discussion

Here we show that exposure of HAZV to elevated [K^+^] causes dramatic structural changes in HAZV glycoprotein spikes, promotes spike–membrane interactions, and expedites infectivity. Taken together, this work suggests that [K^+^] acts as a biochemical cue to trigger the HAZV fusion machinery. As other genetically distinct bunyaviruses, SBV and BUNV, also exhibit the same [K^+^] requirement during entry (25), we propose that the structural changes in response to elevated [K^+^] are a conserved feature common to other bunyaviruses.

HAZV spikes in low [K^+^] are arranged in a 4-fold pattern, appearing as a continuous layer of protrusions from the viral membrane ∼8 nm tall. Therefore, HAZV spikes are similar to those from hantaviruses (15, 29, 32), which is not unexpected because the genetic segments encoding the glycoproteins of hantaviruses and nairoviruses are closely related (2). When exposed to K^+^, the spike arrangement changed extensively. The most radical differences between both conditions were the appearance of a 22-nm long extended spike and loss of the peripheral density surrounding the viral envelope. Large extensions of the Uukuniemi virus spikes were observed under conditions of changing pH rather than K^+^ as described here, with extension from ∼10 nm to ∼18 nm being described (21). Additionally, after HAZV had been treated with high [K^+^], we observed frequent interaction of glycoprotein spikes with co-purified membranes, and we hypothesize that these are mediated by the exposure of the HAZV G_c_ fusion loop. Taken together, these findings are consistent with K^+^ exposure providing a biochemical cue that mediates structural rearrangements and, in the context of infection, enhancing fusion between viral and endosomal membranes to permit genome release. We therefore propose that exposure of HAZV glycoprotein spikes to K^+^ represents a critical step in the HAZV fusion and infection process.

Why the HAZV spike extension is larger than that of the Phenuivirus member Uukuniemi virus is unknown but may result from structural differences between the respective G_n_ and G_c_ proteins. In addition, we cannot rule out the possibility that additional viral or cellular proteins are present within the extended spike density and that these could be derived from the copurifying membranes with which many of the extended spikes are interacting. Furthermore, the relative contribution of G_n_ and G_c_ moieties to the compact and extended spike conformations is unknown because of the resolution achieved in our STA and will require acquisition of higher-quality datasets to resolve this important issue in future studies.

The mechanism by which G_c_ is prevented from premature fusion on RVFV virions was recently revealed to involve shielding by G_n_, with fitting of the crystal structure of G_n_ alongside G_c_ into a cryo-EM reconstruction of the virion (33). At low pH (5.0), the G_n_ shield was repositioned in combination with extension of G_c_ to expose its fusion loop. Although the surface arrangement of nairoviral and phenuiviral glycoproteins are different, these findings offer the possibility that conformations of both HAZV G_n_ and G_c_ may be influenced by the K^+^ trigger, resulting in an upward swing of G_c_ into an upright conformation (Fig. 4), perpendicular to the viral membrane, akin to the erect pre-hairpin intermediate RVFV G_c_ crystal structure (11). Whether this conformational change disrupts a heterotypic G_c_–G_n_ interaction or a homotypic G_c_–G_c_ interaction has yet to be elucidated for nairoviruses.

We showed previously that the infectivity of the model bunyavirus BUNV was enhanced by high [K^+^] (23). Based on our findings here, we propose that this response is similarly induced by radical conformational changes in the glycoprotein spikes. Interestingly, BUNV infectivity enhancement was most effective at a pH of 6.3 compared with 7.3 for HAZV, implying that H^+^ ions also play a role in the priming and fusion process.

The role of pH in the entry process of HAZV has not yet been defined, although our results here suggest that exposure of HAZV particles to a near-neutral pH of 7.3 is not sufficient alone to induce fusion-competent conformational changes in the virus glycoproteins. It may be that the K^+^-mediated conformational changes we observed occur early in the endosomal pathway as the [K^+^] increases and that the additional trigger required for adopting the post-fusion conformation is a decrease in pH. Alternatively, it is possible that the K^+^-induced changes may be pH-independent, in which case K^+^ would represent the first example of a monovalent fusion trigger other that H^+^. In this situation, another trigger (*e.g.* a cellular receptor) might be required because we did not observe fusion events in our cryo-EM experiments.

The dependence of viral fusion on the ionic environment is well documented. In particular, exposure of the fusion machinery to a specific pH alters the protonation state of strategically located pH-sensing residues. The pKa of histidine makes it a prime candidate for performing a pH-sensing role, and its protonation can alter polar interactions with neighboring residues that induce an electrostatic repulsion between mobile interfaces, thus driving conformational changes required for fusion. Several pH-sensing residues have been proposed for Phenuivirus G_c_ proteins, including three histidines in the RVFV G_c_ (34) and two in the SFTSV G_c_ (12). In the case of hantaviruses, a novel pH-sensing mechanism involving an E*X*D motif located seven residues upstream of the fusion peptide has been proposed (14), in which protonation at pH 6.5 led to formation of a carboxylate–carboxylic acid hydrogen bond in the crystal structure. When the D*X*E motif became deprotonated at neutral pH, the organization of the critical fusion loop became disordered. If a similar pH-sensing mechanism exists for nairoviruses, then conformational changes are likely to occur earlier in the endosomal pathway.

In contrast, the mechanism by which K^+^ may mediate the observed conformational changes shown here is not known. Interestingly, the recently solved post-fusion crystal structure of the class II E1 fusion protein of Rubella virus, a member of the Togaviridae family of positive-stranded RNA viruses (19), revealed the coordination of a Ca^2+^ ion. The architecture of this Ca^2+^ binding site appears optimized for cation coordination, and its location between two peptide loops proposed to interact with target membranes is consistent with a critical role of this ion in structural rearrangements relevant to fusion. Furthermore, the substitution of coordinating residues with alanine served to abolish membrane fusion and also infectivity (35). Interestingly, the E1 structure was also solved with a coordinated Na^+^ ion at the same site, although the exposure of Rubella virus to physiologically relevant concentrations of this ion did not provide the same infectivity enhancement as seen with Ca^2+^. It was suggested that membrane insertion and fusion were promoted by distinct ionic triggers, Ca^2+^ and pH, respectively (35). Perhaps for HAZV, K^+^ performs the same role as Ca^2+^, with a coordination site optimized for the monovalent K^+^ cation. Intriguingly, the recently described structure of the Hantavirus G_c_ protein was solved with a bound K^+^ when present under crystallization conditions at high concentrations (14). Whether this K^+^-bound form of G_c_ represents a functionally relevant state is unknown.

Although this work represents the first description of a role for K^+^ in mediating conformational changes in the fusion spike machinery, a critical role for K^+^ in the entry of SNS RNA viruses has recently been described for influenza A virus (IAV), in which exposure of virions to elevated [K^+^] was shown to promote infectivity (36). By performing sedimentation and proteolytic cleavage studies, the role of K^+^ was determined to be in the destabilization of interactions between the IAV genome segments within the internal virion core. Passage of K^+^ through the viral envelope was shown to be mediated by the M2 pore, and destabilization was proposed to expedite entry of the IAV ribonucleoproteins to the cytosol after the fusion process is complete. This requirement is consistent with the rising [K^+^] that occurs with transit through the endosome network and implies that viruses have adopted multiple K^+^-dependent mechanisms to allow uncoating at specific endosome stages. Although we have shown that K^+^ mediates HAZV spike rearrangements, it is possible that additional conformational changes are also induced, possibly within the virion interior. However, for this to occur, the K^+^ must traverse the viral envelope, and our previous work suggests that bunyaviruses do not possesses an ion-permeable channel or pore that would allow this (25).

Consistent with a critical role of [K^+^] in virus infection, we also showed that multiplication of HAZV is prevented by blockade of cellular K^+^ channels that disrupt the endosomal K^+^ gradient. This finding means that we have now confirmed that multiplication of three genetically distinct members of the Bunyavirales order can be blocked in this way (HAZV, SBV, and BUNV), and implies that cellular K^+^ channels may represent a new target for the design of small molecules with broad antiviral activities. In the case of HAZV, susceptibility to K^+^ channel blockade also suggests that the genetically very similar but highly pathogenic CCHFV may also be impeded by this strategy. Cellular K^+^ channels are a proven therapeutic target with multiple examples where clinical pharmacological manipulation has provided effective therapies for channel dysfunction diseases, including hypertension, insomnia, anxiety, and diabetes, and that these drugs may be repurposed as small molecules with antiviral properties is an exciting possibility.

## Experimental procedures

### Cell culture and virus

BHK and A549 cells were grown at 37 °C with 5% CO_2_ in Dulbecco's modified Eagle's medium supplemented with 10% fetal bovine serum, 100 units/ml penicillin, and 100 μg/ml streptomycin. HAZV strain JC280 was used to infect BHK cells at an m.o.i. of 0.01 for 1 h at 37 °C to allow virus entry. After 1 h, noninternalized HAZV was removed and replaced with fresh medium. After 2 days, the medium contacting viral particles was collected for virus purification.

### Virus purification

Harvested growth medium containing HAZV was clarified by filtration. HAZV particles were purified by pelleting through a 20% sucrose cushion, and then the pellets were resuspended in 10 mm Tris buffer (pH 7.3) containing 0.5 mm KCl and 20 mm NaCl and pooled. Infectivity was assessed by plaque assay, and purity was assessed by PAGE, followed by silver staining and Western blotting with anti-HAZV N antiserum.

### Vitrification and visualization by cryo-EM

The resuspended virions were then exposed to high [K^+^] by addition of KCl to a final concentration of 140 mm, followed by incubation at 37 °C for 2 h prior to vitrification by plunge-freezing grids using a Leica EM GP automatic plunge freezer. Cryo-EM was performed using a Tecnai F20 operated at 120 kiloelectron volts. Micrographs were collected using a 4096 × 4096 pixel complementary metal oxide semi-conductor (CMOS) camera.

### Cryo-ET and image processing

Grids for cryo-ET were prepared as described above, with the addition of protein A 10-nm colloidal gold particles (Aurion) to serve as fiducial markers. A Titan Krios microscope operated at 300 kiloelectron volts equipped with an energy-filtered BioQuantum and a K2-summit camera (Gatan) was used to collect single-axis tilt series from −60° to +60° at 2° increments in low-dose mode using Tomography 4 (FEI). Images were acquired in counting mode at a nominal defocus between −5 and −8 μm using an electron dose of ∼ 1 e^−^/Å^2^ (total dose per tilt series was ∼ 70 e^−^/Å^2^). eTOMO (IMOD package (37) was used to calculate 3D reconstructions with a final pixel size of 8.56 Å after binning by a factor of two. The average defocus of individual tomographic projections was calculated using gctf (38), and contrast transfer function (CTF) correction was performed using ctfphaseflip (39).

### Subtomogram averaging

Subtomogram averaging was performed using PEET (40), and basic image processing was done using Bsoft (41). Briefly, ∼4000 subtomograms for low K^+^ conditions were selected semiautomatically by generating a mesh around the viral membrane and using the meshinit program from to calculate initial orientations perpendicular to the viral membrane. A single subtomogram with C4 symmetry imposed was used as initial reference. Translational and rotational alignment of the subtomograms was performed iteratively following PEET's guidelines. At the final stage, duplicated subtomograms and subtomograms with low cross-correlation scores were discarded, resulting in ∼ 1600 subtomograms, and C4 symmetry was applied to the final average. The final resolution was ∼ 25 Å according to the Fourier shell correlation (0.5 cutoff). High K^+^ averages were produced in a similar way, except that ∼250 subtomograms were selected manually (to minimize the possibility of selecting spikes interacting head to head, only spikes from virus–vesicle interfaces or extended spikes not interacting with membranes were selected), and initial orientations were calculated using the spikeInit program. The final average, which contained ∼150 subtomograms, yielded a resolution of ∼45 Å. Subtomograms were visualized using Chimera (42). The volume of the spikes under each condition was estimated in chimera after segmenting them using Segger (43).

### Assessment of HAZV-N protein production

A549 cells were infected with HAZV (m.o.i. = 0.1) for 1 h at 37 °C, and then noninternalized virions were removed. Infected cells were lysed at increasing post-infection time points from 1 to 24 h. N production was assessed by Western blotting.

### Western blot analysis

For preparation of cell lysate following incubation, medium was removed, and cells were washed in ice-cold PBS. 200 μl of ice-cold lysis buffer (5 mm glycerol phosphate, 20 mm Tris, 150 mm NaCl, 1 mm EDTA, 1% Triton X-100, 10% glycerol, 50 mm NaF, and 5 mm Na_4_O_7_P_2_ (pH 7.4)) supplemented with a protease inhibitor mixture was applied to cells, and cell lysates were then removed via cell scraping.

Cell lysates were subsequently resolved by SDS-PAGE, and proteins were transferred from SDS-PAGE gels onto polyvinylidene difluoride membranes via Bio-Rad Trans-blot Turbo. Membranes were probed with a sheep anti-HAZV-N serum prior to addition of the relevant secondary antibodies. Anti-GAPDH antibodies were subsequently used as a loading control. The proteins were visualized using the chemiluminescence (ECL) system, and the film was exposed on an Xograph processor.

### Exposure of HAZV virions to elevated [K^+^]

Exposure of HAZV to high [K^+^] was carried out as previously for BUNV (23). HAZV virions (m.o.i. = 0.1) were diluted 1:11 in buffers containing low or high [K^+^] (by supplementing buffers with 5 mm or 140 mm KCl) at pH 7.3, 6.3, or 5.3 and incubated at 37 °C for 2 h, mimicking the pH decrease and [K^+^] increase along the endocytic pathway. Buffers were prepared on the day of the experiment. pH 7.3 (20 mm Tris) and pH 6.3 (30 mm BisTris) buffers were adjusted to the desired pH using hydrochloric acid and pH 5.3 (50 mm sodium citrate) buffers using citric acid. Control experiments were carried out by virion dilution (1:11) in serum-free medium. Buffers were subsequently diluted out by addition of 2 ml of warm complete Dulbecco's modified Eagle's medium and immediately added to A549 cells. Infected cells were incubated until 18 h post-infection and lysed before SDS-PAGE and Western blot analysis; the antibodies used are outlined above.

## Author contributions

E. K. P., S. H., J. Fontana, J. M., and J. N. B. conceptualization; E. K. P., S. H., R. H., J. Fontana, J. M., and J. N. B. formal analysis; E. K. P., S. H., H. T. B., J. Fuller, J. Fontana, J. M., and J. N. B. investigation; E. K. P., S. H., J. Fuller, J. Fontana, J. M., and J. N. B. methodology; S. H., J. Fontana, J. M., and J. N. B. validation; R. H., J. Fontana, J. M., and J. N. B. supervision; R. H., J. M., and J. N. B. funding acquisition; J. Fontana, J. M., and J. N. B. resources; J. Fontana, J. M., and J. N. B. data curation; J. Fontana software; J. Fontana, J. M., and J. N. B. visualization; J. Fontana, J. M., and J. N. B. project administration; J. M., J. Fontana, and J. N. B. writing-original draft; J. M., J. Fontana, and J. N. B. writing-review and editing.

## Footnotes

2 The abbreviations used are:

SNS: segmented negative-stranded
S: small
M: medium
L: large
N: nucleoprotein
CCHFV: Crimean–Congo hemorrhagic fever virus
HAZV: Hazara virus
BUNV: Bunyamwera virus
cryo-ET: cryo-electron tomography
STA: subtomogram averaging
SFM: serum-free medium
BHK: baby hamster kidney
RVFV: Rift Valley fever virus
TEA: tetraethylammonium
Qd: quinidine
SBV: Schmallenberg virus
GAPDH: glyceraldehyde-3-phosphate dehydrogenase
IAV: influenza A virus.
